# 6q16.3q23.3 duplication associated with Prader-Willi-like syndrome

**DOI:** 10.1186/s13039-015-0151-6

**Published:** 2015-06-25

**Authors:** Laurent Desch, Nathalie Marle, Anne-Laure Mosca-Boidron, Laurence Faivre, Marie Eliade, Muriel Payet, Clemence Ragon, Julien Thevenon, Bernard Aral, Sylviane Ragot, Azarnouche Ardalan, Nabila Dhouibi, Candace Bensignor, Christel Thauvin-Robinet, Salima El Chehadeh, Patrick Callier

**Affiliations:** Laboratoire de Cytogénétique, Plateau Technique de Biologie, CHU de Dijon, Dijon, France; Centre de référence maladies rares « anomalies du développement et syndromes malformatifs » de l’Est, Centre de Génétique, CHU de Dijon, Dijon, France; Laboratoire Biomnis, Lyon, France; Service de Pédiatrie, CH d’Auxerre, Auxerre, France; Service de Pédiatrie, CHU Dijon, Dijon, France

**Keywords:** Complex sSMC, Array-CGH, Prader-Willi-like syndrome, 6q16.3q23.3

## Abstract

**Background:**

Prader-Willi syndrome **(**PWS) is characterized by hypotonia, delayed neuropsychomotor development, overeating, obesity and mental deficiency. This phenotype is encountered in other conditions, defining Prader-Willi-like syndrome (PWLS).

**Case presentation:**

We report a 14-year-old boy with a complex small supernumerary marker chromosome (sSMC) associated with PWLS. The propositus presents clinical features commonly found in patients with PWLS, including growth hormone deficit. Banding karyotype analysis and fluorescence in situ hybridization (FISH) revealed a marker derived from chromosome 6 and a neocentromere as suspected, but array-CGH enabled us to characterize this marker as a der(10)t(6;10)(6qter → 6q23.3::10p11.1 → 10p11.21)*dn*. As far as we know, this is the first diagnosed case of PWLS associated with a complex sSMC, involving a 30.9 Mb gain in the 6q16.3q23.3 region and a 3.5 Mb gain in the 10p11.21p11.1 region. Several genes have been mapped to the 6q region including the *TCBA1* gene, which is associated with developmental delay and recurrent infections, the *ENPP1* gene, associated with insulin resistance and susceptibility to obesity and the *BMIQ3* gene, associated with body mass index (BMI). No OMIM gene was found in the smallest 10p11.21p11.1 region.

**Conclusions:**

We suggest that the duplicated chromosome segment 6q16.3q23.3 may be responsible for the phenotype of our case and may also be a candidate locus of PWLS.

## Background

Prader-Willi syndrome (PWS) is characterized by hypotonia during the neonatal stage and in childhood, accompanied by delayed neuropsychomotor development [1]. Overeating, obesity and mental deficiency arise later on. In addition to obesity, Prader-Willi syndrome includes several other endocrine disorders, such as hypothyroidism, growth hormone deficiency, and hypogonadotropic hypogonadism [1]. PWS is caused by a lack of expression of genes located on paternal chromosome 15q11q13. This lack of gene expression may be due to a deletion in this chromosomal segment, to maternal uniparental disomy of chromosome 15, or to a defect in the imprinting centre on 15q11–15q13, or rarely with small supernumerary marker chromosomes 15 (sSMC) [1]. Small supernumerary marker chromosomes (sSMC) are structurally abnormal chromosomes, generally equal in size to or smaller than a chromosome 20 of the same metaphase spread, that cannot be characterized by conventional banding cytogenetics [2]. sSMC can have different shapes (ring-, centric minute- and inverted duplication-shaped), and in most cases consist of pericentric chromosomal material [3]. They can originate from any part of human chromosomes to form neocentromeres [4]. sSMC are found in 0.043 % of new-borns and in 0.433 % of cases with intellectual disability [5]. A number of other conditions associate obesity and developmental disability. These include UPD14, Cohen syndrome, Bardet-Biedl syndrome, Alstrom syndrome, duplications of 3p25.3–p26.2 and of Xq27.2-ter and deletions 1p36, 6q16 (*SIM1*), and 10q26 [6]. A recent study by array-CGH in 100 children with syndromic obesity showed new candidate genes (*PLIN2, CDH13, CNTNAP2, CPPED1*, *NDUFA4, PTGS2* and *SOCS6*) and 22 % of patients with pathogenic or potentially pathogenic CNVs were identified [7].

We report a boy with PWLS associated with a small supernumerary marker chromosome (sSMC). This is the first case of an sSMC involving chromosome 6 and 10 and was characterized as der(10)t(6;10)(6qter → 6q23.3::10p11.1 → 10p11.21)*dn*. To the best of our knowledge, this is the first PWLS which could be due to a complex sSMC.

### Case presentation

**Fig. 1:**
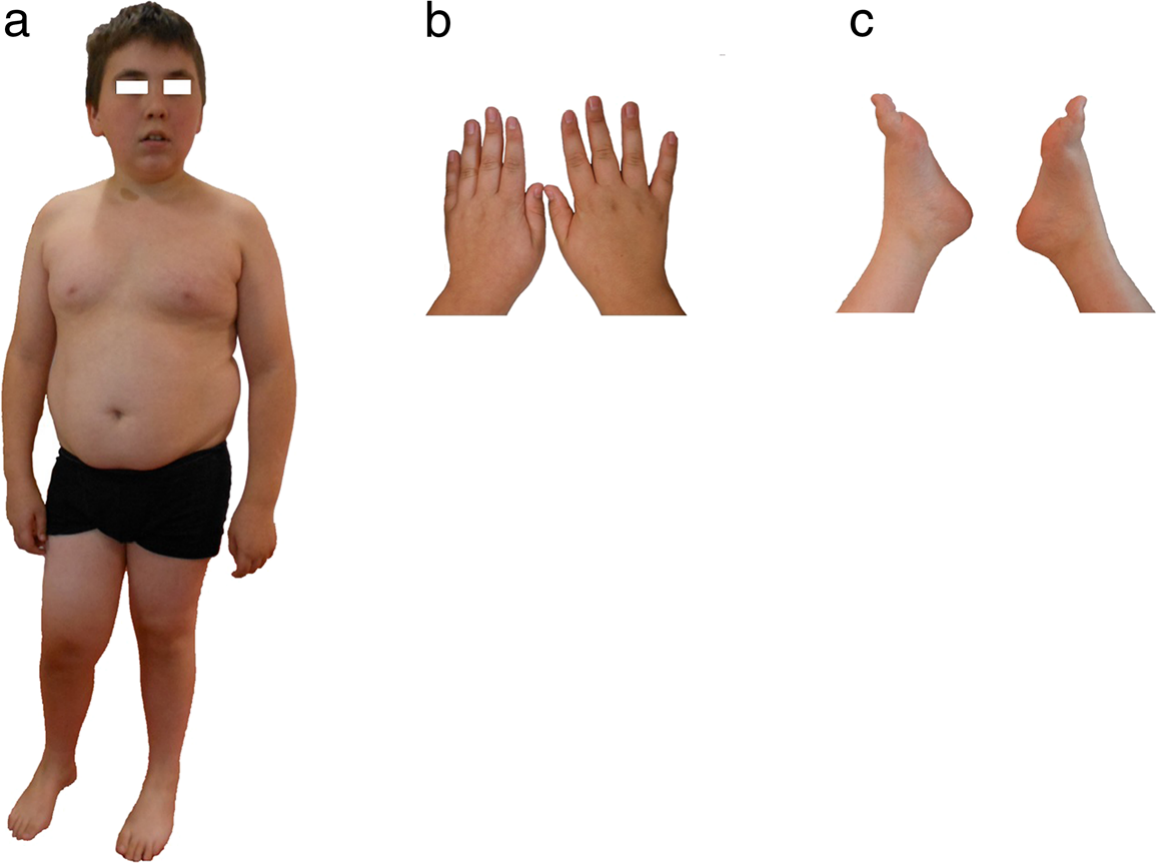
Phenotype of the patient at the age of 15 years. **a**. Note the central obesity, short stature, café-au-lait spots and facial features of Prader-Willi. **b** and **c**. Small hands and feet

The patient, a male, was born to healthy, non-consanguineous parents and has 2 sisters. The heights of the father and mother are 174 and 156 cm, respectively. The patient was born at full term; his birth weight was 3610 g, length 52.5 cm, head circumference 35 cm. Several respiratory tract infections were treated during his first years of life and saturnism was diagnosed at 5 years old, with no need for treatment. The patient had normal psychomotor acquisition (walked at 16 months), developmental delay with school retardation and speech articulation defects. At 15.5 years old (Fig. 1a), the patient was 145.4 cm tall (−3.8SD), 55.3 kg in weight (BMI = 26.6 kg/m^2^ = +2.8SD: overweight according to WHO criteria [8]) suffered from intellectual deficiency and could neither read nor write. He had central obesity, small hands and feet (Fig. 1b, c), short stature, pubertal delay (small penis and testes), lower limb livedo, café-au-lait spots on his neck and the facial features of Prader-Willi syndrome. These observations were compatible with the Prader-Willi-like phenotype [6, 9]. Bioassays showed isolated GH deficiency with low IGF-I concentrations with no evidence of hypothalamic-pituitary disease with normal MRI.

## Methods

Blood samples of the patient and his parents were drawn after informed consent had been obtained. Chromosome analysis was performed on 72-h lymphocyte cultures according to standard techniques [10]. Preparations were GTG and RHG banded by standard procedures. Fluorescence in situ hybridization (FISH) was carried out using octochrome cytocell® according to the manufacturer’s instructions (Cytocell Ltd, Cambridge, United Kingdom). This kit combines an 8-square multiprobe device and the whole chromosome painting probe (labelled in 3 different colours) to allow all 24 chromosomes to be identified on a single slide. Array-CGH analysis was performed according to the Agilent protocol with minor protocol modifications using Agilent 4x180K [11].

## Results

**Fig. 2:**
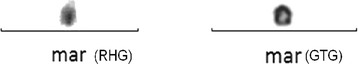
sSMC on Karyotype RHG and GTG banding

**Fig. 3:**
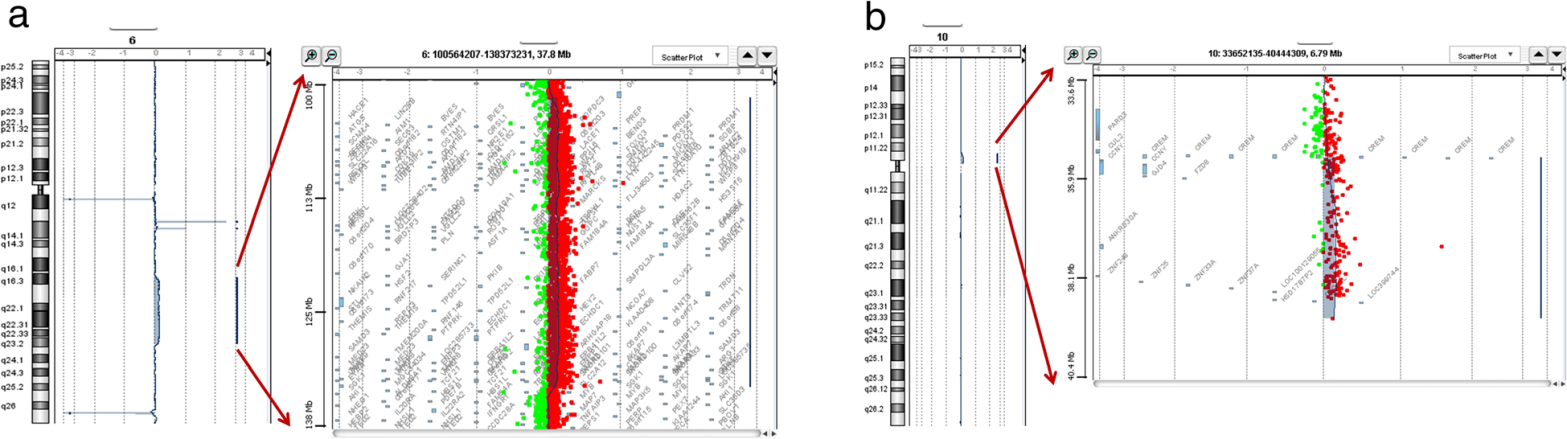
Result of the array-CGH genotyping (GRCh37/hg19) of the genomic gain, originating from chromosome 6 (**a**) and 10 (**b**). **a**. The array-CGH showed a 30.9 Mbp gain in the 6q16.3 - q23.2 region (**b**). The array-CGH showed a 3.5 Mbp gain in the 10p11.21 - p11.1 region

**Fig. 4:**
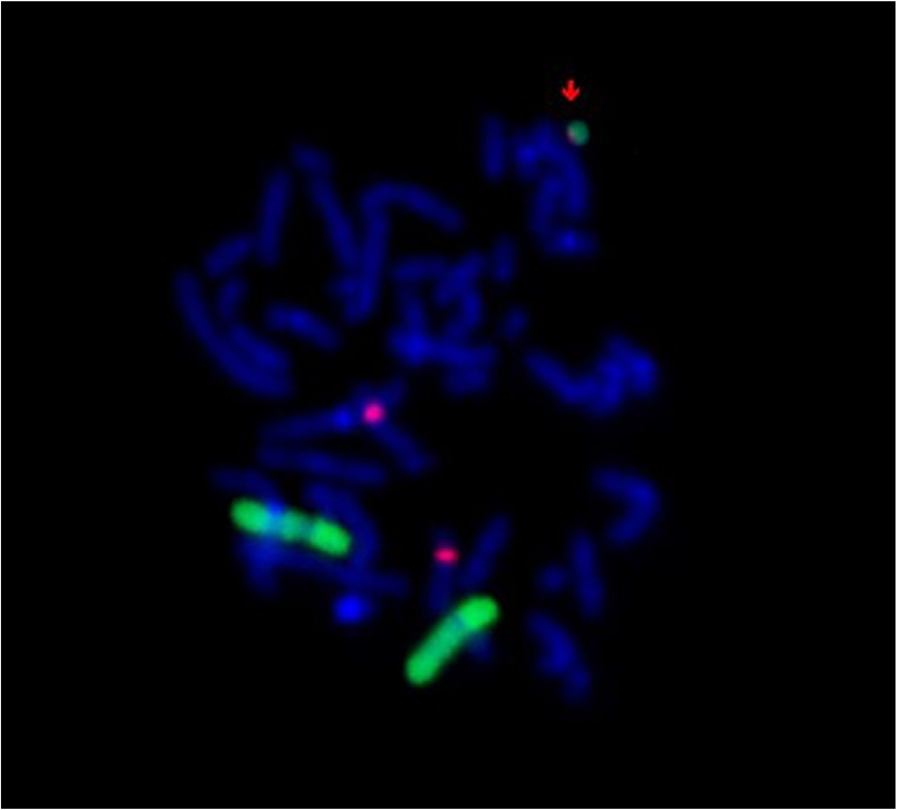
The FISH result with whole painting of chromosome 6 and in red the centromere probe of chromosome 10. The complex sSMC is doubly labelled

Conventional cytogenetic analysis on cultured T-lymphocytes revealed a male karyotype with a small supernumerary marker chromosome in 32 % of the cells analysed: mos 47, XY,+mar[8]/46,XY[17] (Fig. 2). Parental karyotypes from peripheral blood lymphocytes were both normal, indicating a *de novo* origin of the sSMC. The karyotype after conventional cytogenetic analysis and FISH (whole painting probe) can be described as: mos 47,XY,+mar[8]/46,XY[17].ish neo(6)(wcp6+, D6Z1-, tel6p-, tel6q-)dn. Array-CGH analysis confirmed the duplicated region of chromosome 6 (Fig. 3a): 6q16.3q23.3 (102,474,305-133,386,766) of 30.9 Mb that included the 153 genes previously observed in FISH and showed a duplicated pericentromeric region 10p11.21p11.1 (35,490,990-39,076,732) (GRCh37/hg19) of 3.5 Mb that included 7 genes (Fig. 3b). The duplicated region of chromosome 10 was confirmed by another centromere FISH of chromosome 10 (Fig. 4). The resulting karyotype associated with array-CGH analysis can be described as 47,XY,+ der(10)t(6;10)(6qter → 6q23.3::10p11.1 → 10p11.21)*dn* arr[hg19]6q16.3q23.3(102,474,305-133,836,766)×2 ~ 3,10p11.21p11.1(35,490,990-39,076,732)×2 ~ 3. Array-CGH mosaicism of chromosome 10 and chromosome 6 was estimated at about 25.0 % (log2 ratio: 0.14). The mosaicism rate found with array-CGH analysis was similar to that found with karyotyping. Maternal uniparental disomy of chromosome 15 was ruled out (data not shown).

## Discussion

To the best of our knowledge, this is the first report of a patient with PWLS associated with a complex sSMC, which involved chromosome 6 and 10 at breakpoints of 6q16.3q23.3 and 10p11.21p11.1, respectively. Complex small supernumerary marker chromosomes (sSMC) are a subgroup of sSMC derived from more than one chromosome [2]. The Liehr sSMC database [12] has collected approximately 412 cases of complex sSMC from the literature, which represent 8.4 % of all sSMC cases [4]. The majority of complex sSMC cases are der(22)t(11;22)(q23;q11.2) or Emanuel syndrome cases (339/412 cases; 82 %) and present generally in banding cytogenetics as centric minutes [3]. Among the 73 complex sSMC described by Liehr T. excluding Emanuel syndrome, 64 % were inherited from a balanced translocation in one parent (83 % maternally derived) [3] and 10 % were in mosaic. All of the human chromosomes, except chromosome 10, have already been shown to be involved in complex sSMC [3].

To investigate whether the duplicated 6q16.3q23.3 and 10p11.21p11.1 regions could play a role in PWL syndrome, all cases with comparable chromosome 6q and 10p duplications documented in PubMed, the sSMC Liehr database [12] and DECIPHER [13] were reviewed. Duplications of the 6q16.3q23.3 region are rare and no similar cases with sSMC comprising the 6q16.3q23.3 region have ever been described in the literature [12, 14]. Two cases with 6q16.3q23.3 duplications are reported in the DECIPHER database [13] (available from http://decipher.sanger.ac.uk and via email from decipher@sanger.ac.uk.) with the phenotype: case 4145 (7 Mb duplication) with brachycephaly, epicanthus, intellectual disability, intrauterine growth retardation, muscular hypotonia and ptosis; case 2068365 (2.3 Mb duplication) with hydrocephalus, macrocephaly and parietal foramina. Three cases with 6q21q23 duplications were described only at the cytogenetic level and associated with developmental delay, congenital hearts defects, depressed nasal bridge and epicanthal folds [15, 16] and two cases of 6q21q22.1 in a mother and her daughter showed similar phenotypes (cognitive difficulties, obesity, essential tremor) and café-au-lait spots in the daughter [17]. Regarding other regions of 6q chromosome, trisomy 6qter has also been associated with mental retardation and obesity starting from childhood [18, 19], but cases of neocentric sSMC (6q) phenotypes show no similarity to ours [12, 20, 21]. Our patient’s complex sSMC involves chromosome 10 too and it is, to the best of our knowledge, the first report of a complex sSMC involving chromosome 10 and characterized by array-CGH. All human chromosomes have already been included in a complex sSMC except for chromosome 10. The pericentromeric region of chromosome 10 is known to comprise many copy number variations and other repetitive elements [22]. In our case, the OMIM gene map search [23] for the duplicated 10p11.21p11.1 region revealed no disease-causing genes.

A total of 60 CNVs have been found in 100 patients with syndromic obesity in a recent study [7] using array-CGH and can be classified into three groups: the first was considered clinically relevant for syndromic obesity (*n* = 14), the second potentially relevant (*n* = 17) and the third probably benign (*n* = 29). The second group with potentially clinically relevant CNVs (inherited from one parent with an abnormal phenotype) contained genes of particular interest involved in insulin or adiponectin receptor and peroxisomal acid metabolism: *SOC6, PLIN2, CDH13, CNTNAP2, SOX3* and *ACOXL*.

The OMIM gene map search [23] of the duplicated 6q16.3q23.3 region revealed more than 150 genes including *TCBA1*, *BMIQ3* and *ENPP1* genes. Two balanced translocations showed that the *TCBA1* (*NKAIN2*; Na+/K+ transporting ATPase-Interacting Protein 2) gene was truncated in 1 patient with a severe neurological phenotype (epileptic encephalopathy with spastic tetraparesis and severe psychomotor retardation, microcephaly, hand dysmorphism and hypogonadism with micropenis and cryptorchidism), and a second patient with developmental delay and recurrent infections [24, 25]. These two de novo translocations provide evidence that constitutional inactivation (haploinsufficiency) of the *TCBA1* gene causes developmental delay and a distinct phenotype. *BMIQ3* was linked with body mass index (BMI) using a genome-wide linkage analysis [26] using polymorphic markers and LOD scores. In the same way, *ENPP1* was associated with insulin resistance and susceptibility to obesity [27]. Moreover, Prader-Willi-like syndrome with growth hormone deficit has been described with 6q16.1–q21deletions [28, 29] and associated with Single-minded 1 gene (*SIM1*) at 6q16.2 [30] and/or *POU3F2* (*BRN2*) at 6q16.1 [29]. However the *SIM1* and *POU3F2* genes were not included in the duplicated 6q16.3q23.2 region of our patient.

## Conclusions

According to these data and a review of the literature, our patient’s phenotype seems to be related to the euchromatin of chromosome 6 rather than chromosome 10. The duplicated 6q16.3q23.3 may be a candidate locus of PWLS with *TCBA1* as the candidate gene for mental retardation and *BMIQ3* and *ENPP1* implicated in the susceptibility to obesity. This case illustrates the importance of array-CGH to characterize more precisely sSMC not elucidated by FISH, and suggests that all neocentromere markers detected by karyotype must by analysed by array-CGH.

## Consent

Written informed consent was obtained from the patient’s parents for publication and accompanying images of this case report. A copy of the written consent is available for review by the Editor-in-Chief of this journal.
